# *Mycobacterium tuberculosis* Infection in Young Children: Analyzing the Performance of the Diagnostic Tests

**DOI:** 10.1371/journal.pone.0097992

**Published:** 2014-05-30

**Authors:** Tomàs M. Pérez-Porcuna, Carlos Ascaso, Adriana Malheiro, Rosa Abellana, Marilaine Martins, José Felipe Jardim Sardinha, Patricia Quincó, Irineide Assumpção Antunes, Marlucia da Silva Garrido, Samira Bührer-Sékula, Flor Ernestina Martinez-Espinosa

**Affiliations:** 1 Pós-Graduação em Medicina Tropical, Universidade do Estado do Amazonas/Fundação de Medicina Tropical Dr. Heitor Vieira Dourado, Manaus, Amazonas, Brazil; 2 Departament de Salut Pública, Facultat de Medicina, Universitat de Barcelona, Barcelona, Catalunya, Spain; 3 Servei de Pediatria, CAP Valldoreix, Unitat de Investigació Fundació Mútua Terrassa, Hospital Universitari Mútua Terrassa, Terrassa, Catalunya, Spain; 4 Institut d’Investigacions Biomèdiques August Pi i Sunyer, Barcelona, Catalunya, Spain; 5 Laboratório de Imunologia Básica e Aplicada, Fundação de Hematologia e Hemoterapia do Amazonas, Manaus, Amazonas, Brazil; 6 Universidade Federal do Amazonas. Manaus, Amazonas, Brazil; 7 Fundação de Medicina Tropical Dr. Heitor Vieira Dourado, Manaus, Amazonas, Brazil; 8 Centro de Referência da Tuberculose, Policlínica Cardoso Fontes, Manaus, Amazonas, Brazil; 9 Programa de Controle da Tuberculose, Departamento de Vigilância Epidemiológica, Fundação de Vigilância em Saúde do Estado do Amazonas, Manaus, Amazonas, Brazil; 10 Instituto de Patologia Tropical e Saúde Pública, Universidade Federal de Goiás, Goiânia, Goiás, Brazil; 11 Instituto Leônidas e Maria Deane - Fiocruz Amazônia, Fundação Oswaldo Cruz, Manaus, Amazonas, Brazil; Fundació Institut d’Investigació en Ciències de la Salut Germans Trias i Pujol. Universitat Autònoma de Barcelona. CIBERES, Spain

## Abstract

**Objective:**

This study evaluated the performance of the Tuberculin Skin Test (TST) and Quantiferon-TB Gold in-Tube (QFT) and the possible association of factors which may modify their results in young children (0–6 years) with recent contact with an index tuberculosis case.

**Materials and Methods:**

A cross-sectional study including 135 children was conducted in Manaus, Amazonas-Brazil. The TST and QFT were performed and the tests results were analyzed in relation to the personal characteristics of the children studied and their relationship with the index case.

**Results:**

The rates of positivity were 34.8% (TST) and 26.7% (QFT), with 14.1% of indeterminations by the QFT. Concordance between tests was fair (Kappa = 0.35 *P*<0.001). Both the TST and QFT were associated with the intensity of exposure (Linear OR = 1.286, *P* = 0.005; Linear OR = 1.161, *P* = 0.035 respectively) with only the TST being associated with the time of exposure (Linear OR = 1.149, *P* = 0.009). The presence of intestinal helminths in the TST+ group was associated with negative QFT results (OR = 0.064, *P* = 0.049). In the TST− group lower levels of ferritin were associated with QFT+ results (Linear OR = 0.956, *P* = 0.036).

**Conclusions:**

Concordance between the TST and QFT was lower than expected. The factors associated with the discordant results were intestinal helminths, ferritin levels and exposure time to the index tuberculosis case. In TST+ group, helminths were associated with negative QFT results suggesting impaired cell-mediated immunity. The TST−&QFT+ group had a shorter exposure time and lower ferritin levels, suggesting that QFT is faster and ferritin may be a potential biomarker of early stages of tuberculosis infection.

## Introduction

Tuberculosis (TB) is one of the infectious diseases producing the greatest morbidity and mortality worldwide [1]. Children represent 5 to 15% of the cases around the world and are more frequently infected and more easily affected and have the most severe forms of the disease [2].

Young children are mainly infected in the domiciliary setting by a household-sharing adult patient [3]. The main strategy of control of TB at a pediatric level is the detection of cases in the study of contacts and early treatment of the infection or disease. Diagnosis of infection is fundamental to implement this strategy [4].

The diagnosis of *Mycobacterium tuberculosis* (*Mtb*) infection is complex due to the lack of a gold standard [5]. The study of infection is based on the evaluation of immune response to the exposure of typical antigens of *Mtb* [5]. For decades the Tuberculin Skin Test (TST) has been the main diagnostic method of *Mtb* infection. It is based on the measurement of cell-mediated immunity expressed through delayed-type hypersensitivity response after subepidermic administration of purified protein derivatives (PPD) of *Mtb* [6]. The interpretation of the TST has been questioned especially because of the possibility of false positives in populations with high Calmette-Guérin bacillus vaccine (BCG) coverage and non-tuberculous mycobacteria (NTM) exposure [7]. In the last years new diagnostic techniques such as Interferon Gamma Release Assays (IGRAs) have been developed to improve the diagnosis of *Mtb* infection. The IGRAs are based on *in vitro* measurement of interferon gamma (IFN-γ) in the response of T lymphocytes versus the *Mtb*-specific region of difference 1 (RD1) antigens, which are very specific, albeit not exclusive, of *Mtb* [8]. Numerous studies have been performed to analyze these techniques but relatively few have assessed the performance of these tests in small children [9].

The aim of this study was to evaluate the response of the IGRA QuantiFERON-TB Gold In-Tube (QFT) and TST tests in young children with recent exposure to an index case. In addition, we evaluated the factors which may modify test results, with special emphasis on those which alter the immunity of children in a population systematically vaccinated with BCG in the perinatal period.

## Materials and Methods

### Ethics Statement

The study was approved by the Ethical Committee in Investigation of the Fundação de Medicina Tropical Dr. Heitor Vieira Dourado (FMT-HVD), October 26, 2007 (Protocol 2865-07). All legal guardians of participants provided written informed consent for inclusion in the study. All the individuals included in the studied received adequate treatment according to their clinical status.

### Setting and Study Population

We conducted a cross-sectional study of comparison of diagnostic tests.

Case recruitment was performed in the Policlínica Cardoso Fontes (regional reference center for TB) and in the FMT-HVD, Manaus, Amazonas, Brazil from March 2009 to February 2010. The adults (greater than 12 years of age) diagnosed with tuberculosis in both centers were questioned about contact with children from 0 to 6 years of age. Those responding affirmatively were invited to bring the children to the center for evaluation and to be invited to participate in the study. All the adult index cases were sputum smear and/or culture positive.

We included children from 0–6 years of age with recent contact with an adult symptomatic TB index case within the last 12 months. Subjects receiving treatment or prophylaxis for TB were excluded. The study was undertaken at an outpatient level.

A sample size of 97 individuals was calculated for the study of concordance between diagnostic tests with a confidence level of 95%, precision of 15%, an expected kappa coefficient of 0.7 and with an expected proportion of positive classified by the diagnostic tests of 35%. These calculations were performed with the EPIDAT 3.1 program (Consellería de Sanidade, Xunta de Galicia, Spain and Pan American Health Organization, Washington D.C., USA).

### Data Collection

Demographic data, epidemiologic history of exposure to an index case of TB, the clinical history of the patient and physical examination were recorded. Chest X-ray, TST, stool and blood analysis (prior to the TST) were performed. The human immunodeficiency virus (HIV) test was not obligatory but was recommended to all the participants.

The *Mycobacterium tuberculosis* contact score (MTC-score) was used to evaluate the intensity of exposure [10]. The MTC-score is from 0 to 15 and is based on the assumption that the gradient of *Mtb* exposure is a composite function of the infectivity of the index case (0–4), the duration of exposure hours per day (0–4), the relationship to the index case (0–4) and the type of exposure (0–3) [10].

The time (month) from symptom onset to the time of initiation of treatment of the index case was calculated to measure the total exposure time (contagion) of the child to the index case.

Nutritional evaluation: The weight of all the study subjects was obtained and the standard deviation (SD) of the weight was calculated based on age and sex with the Anthro programme (WHO, version 3.2.2, January 2011). The risk of malnutrition was defined as gender-specific weight-for-age less than 1 SD and malnutrition was determined as less than 2 SDs [11].

### Procedures

The TST was performed with an intradermic injection of 2 tuberculin units (TU) of PPD RT23 (Statens Serum Institut, Copenhagen, Denmark) and read 72 hours thereafter. A strong TST reaction (TST+) was defined when induration was ≥10 mm according to the protocols of the World Health Organization (WHO) [4].

### Diagnostic Laboratory Tests

#### Blood tests

The QFT (Cellestis, Carnegie, Australia) was carried out and interpreted according to the manufacturer’s instructions by experienced laboratory technicians who were unaware of the data of the study subjects. The result of the test was considered positive (QFT+) if the net value of IFN-γ to the TB antigens (after subtracting the negative control) was ≥0.35 IU/mL and ≥25% of the value of the negative control, independently of the response of the mitogen. The result of the test was considered negative (QFT−) if the net value of the IFN-γ was <0.35 IU/mL and mitogen response was sufficient (≥0.50 IU/mL). A test result was considered indeterminate if there was excessive IFN-γ production with the negative control tube ≥8.0 IU/mL (indeterminate hypereactive) or with insufficient net mitogen response <0.50 IU/mL plus insufficient net response of the TB antigen <0.35 IU/mL (indeterminate hyporeactive). When the QFT result was indeterminate the test was repeated to confirm the result. Quantitative determination of 25-OH vitamin D and ferritin was made by immunoassay kits (LIAISON 25 OH Vitamin D Total and LIAISON Ferritin, DiaSorin, Saluggia, Italy).

#### Stool collection and examination

Three stool samples were collected from each child on three consecutive days in containers with a wide mouth screw-cap containing 10% formalin. Detection of eggs was made by the spontaneous sedimentation method [12].

### Statistical Analysis

Description of categorical variables was performed using frequency tables, and quantitative variables were determined using medians, means, and interquartile range (IQR). To identify variables associated with the results of the tests bivariate analysis was first performed with the Fisher exact or Chi-square tests for categorical variables, and the Mann Whitney U test was used for quantitative variables followed by multiple logistic regression analysis. The independent variables analyzed were: sex, age, weight by age and sex, corticotherapy or vaccination within the previous 3 months, BCG, passive smoking, characteristics of the index case, characteristics of the child and vitamin D and ferritin levels and the presence of intestinal helminths.

To analyze the discordances between the TST and QFT results multivariate logistic regression was carried out and stratified on the basis of the results of the TST to know the differential behavior of the QFT. Associations between categorical independent variables and the test result are expressed as odds ratio and otherwise as linear odds ratios. The linear odds ratio quantifies the magnitude of the association between the positive response of the test and the change that occurs to a unit increase/decrease in the quantitative variable of interest. To show this effect, a plot of the odds ratio and its confidence interval for each value of levels of the variable was created. The y axis scaled is the same for all the plots.

Concordance between tests was measured using the Kappa index [13]. The receiver operating characteristic (ROC) curve was used in the TST negative (TST−) group to determine a cut-off of ferritin diagnosis between the QFT positive and negative (QFT+ and QFT−) populations. The type I error was set at 5%. The analyses were performed using the statistical package PASW Statistics 18 (SPSS Inc., Chicago, IL, USA).

## Results

### Descriptive

**Figure 1:**
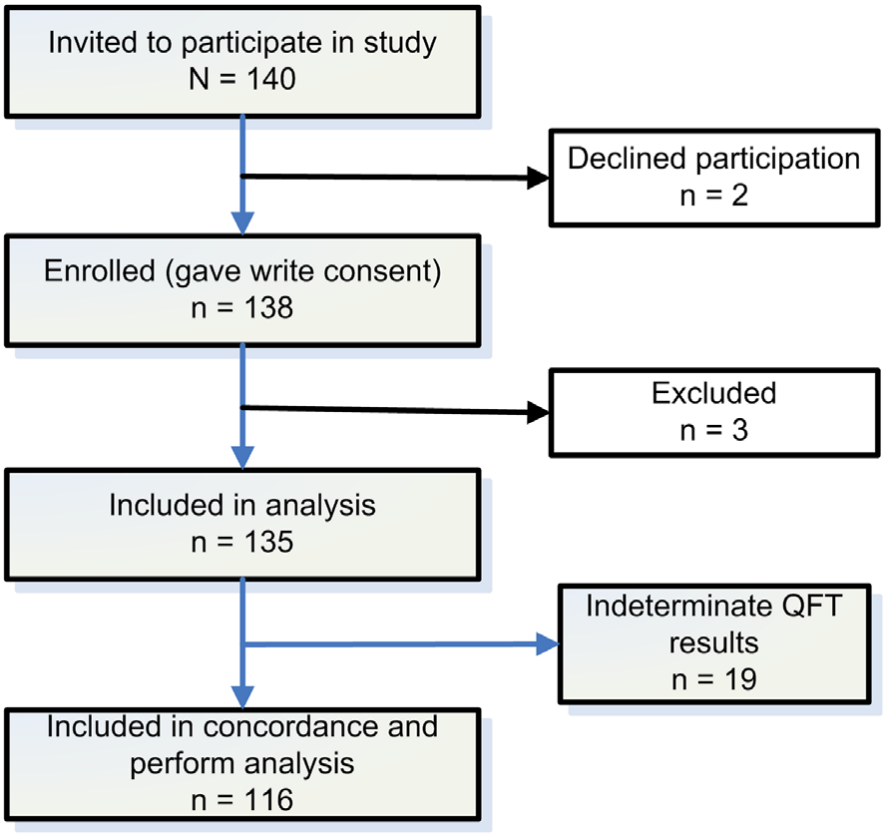
Flow diagram of enrolment (QFT: QuantiFERON-TB Gold In-Tube).

We evaluated 140 children for the study of the comparison of the TST and QFT results. Of these, 2 children refused to participate and 3 dropped out and thus, 135 were finally included in the study (Figure 1).

The median age of the participants was 46 months (IQR: 28.0–64.5), with 23.0% being of less than 24 months; 54.8% (74/135) were girls and 90.8% (118/130) presented the BCG scar. Contact with an intrahousehold index TB case occurred in 77.8% (105/135) of the children. The adult source case was smear positive in 45.2% (61/135) and the median MTC-score was 11 (IQR: 6.3–13.0) and the median time of exposure was 1.5 months (IQR: 0.2–3.7). The median ferritin level of the participants was 31.9 ng/mL (IQR: 21.6–45.9). Risk of malnutrition or status of malnutrition was presented by 23.3% (30/129) of the children, intestinal helminths (*Ascaris lumbricoides* and/or *Trichuris trichiura*) were present in 28.6% (30/105) and 33.8% (44/130) were passive smokers (Table 1).

**Table 1:** Baseline demographic and clinical data of the study participants.

	Individuals evaluated	All Results#
**Age (months)**	135	46 (28.0; 64.5)
Less than 24 months		31 (23.0%)
**Gender**	135	
Male		61 (45.2%)
Female		74 (54.8%)
**BCG scar**	130	118 (90.8%)
**BCG vaccination card**	133	130 (97.7%)
**Household contact**	135	105 (77.8%)
**Adult smear positive**	135	61 (45.2%)
**Time of exposure (months)**	135	1.5 (0.2–3.7)
**MTC-score**	135	11 (6.3; 13.0)
**Passive Smokers**	130	44 (33.8%)
**Previous viral infection** a	135	28 (20.7%)
**Previous vaccination (not BCG)** a	135	71 (52.6%)
**Previous corticoid therapy** a	135	12 (8.9%)
**Helminth infection**	105	30 (28.6%)
**Z scores forweight-for-age and gender**	129	−0.13 (−0.9; 0.5)
**Nutrition Status**	129	
Risk of malnutrition^b^		19 (14.7%)
Malnutrition^b^		11 (8.5%)
**Ferritin level (ng/mL)**	135	31.9 (21.6; 45.9)
**Vitamin D level (ng/mL)**	135	38.1 (24.6; 48.8)
**TST**	135	
Strong reaction (≥10 mm)		47 (34.8%)
Weak reaction (5–9 mm)		0 (0.0%)
No reaction (0–4 mm)		88 (65.2%)
**QFT result**	135	
Positive		36 (26.7%)
Negative		80 (59.3%)
Hyperactivity of the negative control		10 (7.4%)
No reactivity of the positive control		9 (6.7%)

TST: Tuberculin Skin Test, QFT: QuantiFERON-TB Gold In-Tube, MTC-score: *Mycobacterium tuberculosis* contact score, BCG: Bacillus Calmette-Guérin.

# Categorical variables expressed as number of subjects (n) and percentage (%) compared to those evaluated with the characteristic studied. Quantitative variables expressed as mean and interquartile range (IQR).

a In the 12 weeks prior to the study.

b Risk of malnutrition was defined as a Z score for weight less than −1 SDs for age and gender; malnutrition was defined −2 SDs for age and gender.

The TST showed a strong reaction (≥10 mm) in 34.8% (47/135) of the children, a weak reaction (5–9 mm) in 0% (0/135) and no reaction (0–4 mm) in 65.2% (88/135). The QFT was positive in 26.7% (36/135) of the children, negative in 59.3% (80/135) and indeterminate in 14.1% (19/135) by hyperactivity of the negative control in 7.4% (10/135) [excessive background IFN-γ production] and no reactivity of the positive control in 6.7% (9/135) [insufficient IFN-γ response to mitogen]. Concordance between the TST and QFT (discarding the indeterminate cases) was fair, with a Kappa index of 0.350 (*P*<0.001) (Tables 1, 2).

**Table 2:** Comparison of the TST and QFT results.

		QFT results				
		Positive	Negative	Indeterminate	Indeterminate	Total
				Hyporeactive	Hyperreactive	n (%)
**TST results**	Positive	21	18	2	6	47 (34.8%)
	Negative	15	62	7	4	88 (65.2%)
	Total n (%)	36 (26.7%)	80 (59.3%)	9 (6.7%)	10 (7.4%)	135 (100.0%)

TST: Tuberculin Skin Test, QFT: QuantiFERON-TB Gold In-Tube, n: number of subjects.

The samples required to perform the analysis of intestinal helminths were not available in 24.1% of the children. These missing data were not associated with the results of either the TST or the QFT (chi-squared = 1.055 and *P* = 0.788), and the missing data percentages for the four combinations of the test results ranged between 20% and 33%.

### General Concordance and Analysis of the Test Responses

The results of each test were analyzed taking into account the different demographic, epidemiologic and clinical variables of the children included in the study.

**Figure 2:**
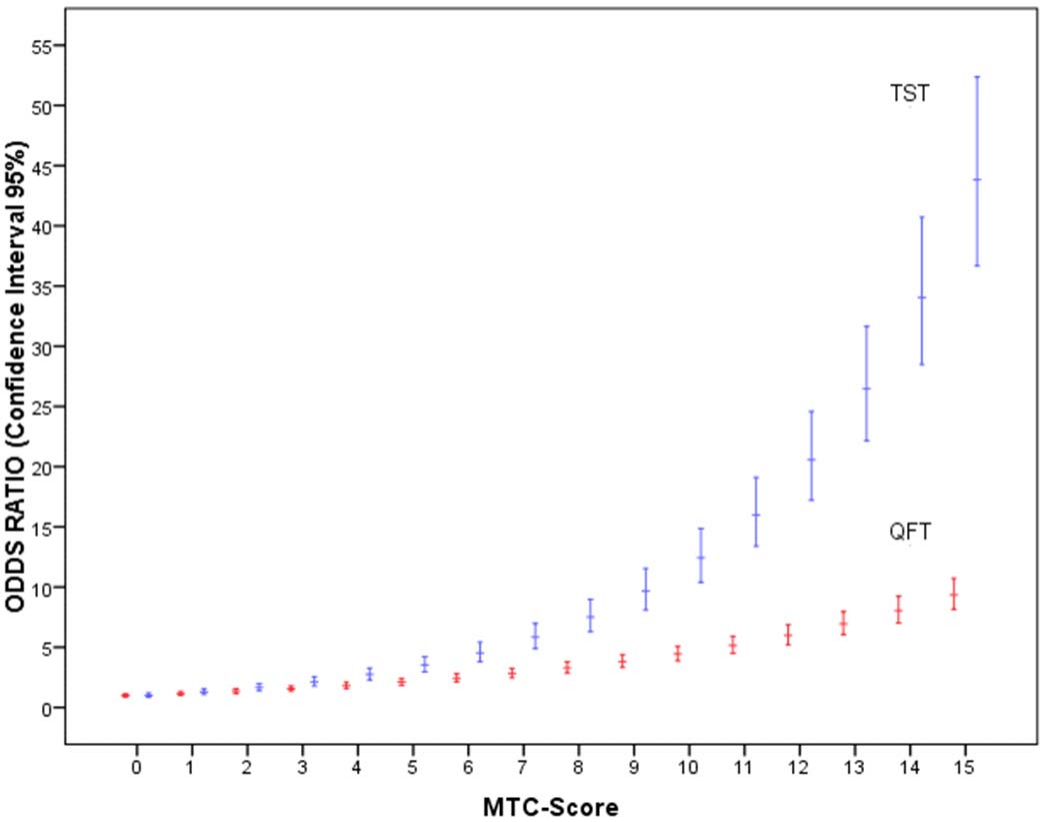
Odds ratio and confidence interval of 95% for the Tuberculin Skin Test –TST− (blue) and QuantiFERON-TB Gold in-Tube –QFT− (red) positive results according to the intensity of exposure to index case by *Mycobacterium tuberculosis* contact score (MTC-score). The MTC-score is from 0 to 15 and is based on the assumption that the gradient of *Mtb* exposure is a composite function of the infectivity of the index case (0–4), the duration of exposure -hours per day- (0–4), the relationship to index case (0–4) and the type of exposure (0–3).

**Figure 3:**
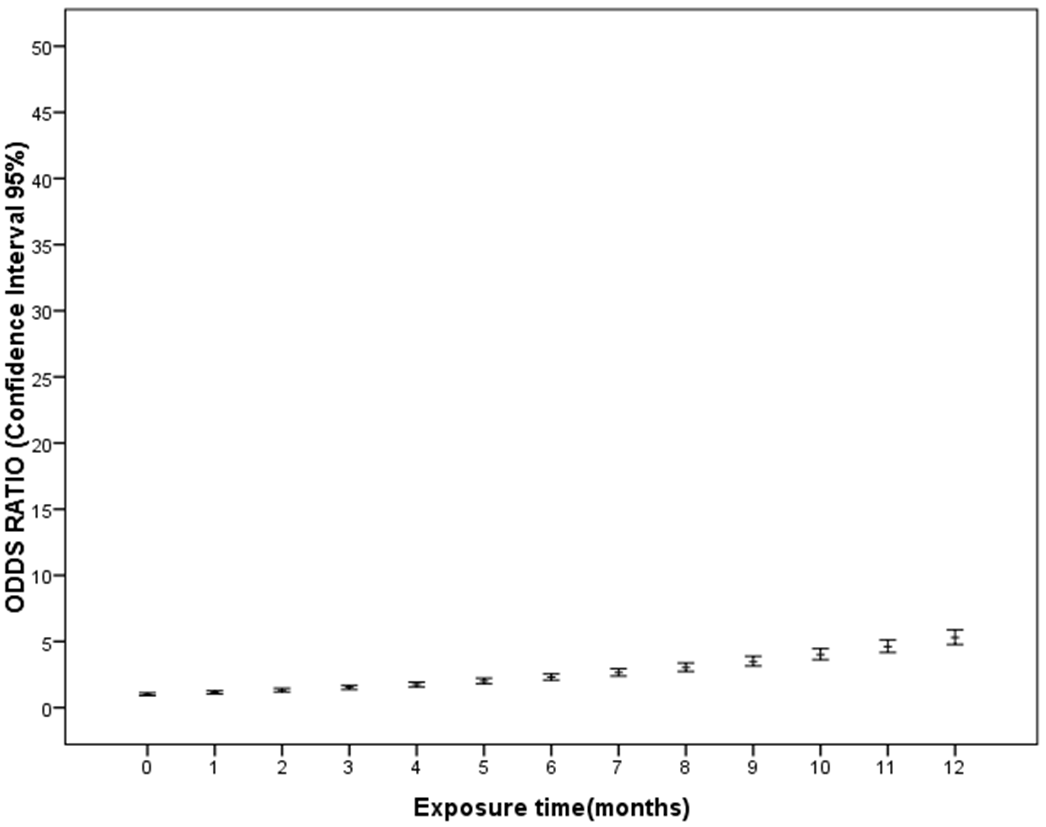
Odds ratio and confidence interal of 95% for the Tuberculosis Skin Test positive results according to time of exposure (months) to index case.

Bivariate analysis of the TST+ respect to TST− results showed an association with a greater level of exposure -MTC-score- (U = 796.0 *P*<0.001), greater time of exposure (U = 581.0 *P*<0.001) and with being a passive smoker (chi-square = 5.6 *P* = 0.017). The multivariate logistic regression model indicated that the TST depended on the time of exposure (Linear OR = 1.149; [95% Confidence Interval (CI): 1.036–1.274]) and the MTC-score (Linear OR = 1.286; CI: 1.077–1.536) (Table 3 and Figures 2, 3).

**Table 3:** Bivariate analysis and Multivariate logistic regression for TST results and QFT results.

	TST results					QFT results				
	Bivariate			Multivariate		Bivariate			Multivariate	
	Positive#	Negative#	*P* value	*P* value	Odds Ratio	Positive#	Negative#	*P* value	*P* value	Odds Ratio
	N = 39	N = 77			(95% CI)	N = 36	N = 80			(95% CI)
Age (months)	45.0	46.0	0.863	0.729	-	47.5	44.0	0.241	0.412	-
	(26.0, 60.0)	(24.0, 65.0)				(32.0, 66.5)	(23.0, 62.0)			
Gender (male)	46.2%	40.3%	0.544	0.906	-	41.7%	43.2%	0.876	0.665	-
BCG scar	94.9%	90.5%	0.418	0.244	-	97.2%	89.7%	0.169	0.560	-
Time of exposure (months)	4.0	1.0	<0.001	0.009	1.15^b^	2.0	1.0	0.024	0.537	-
	(1.0, 10.0)	(0.0, 3.0)			(1.04; 1.27)	(1.0–5.2)	(0.0–3.0)			
MTC-score	12.0	10	<0.001	0.005	1.29^b^	12.0	10.0	0.021	0.035	1.16^b^
	(8.2, 14.0)	(0.0,12,0)			(1.08; 1.54)	(8.0, 14.0)	(0.0, 12.0)			(1.01; 1.33)
Passive Smokers	50.0%	27.4%	0.017	0.323	-	41.7%	32.9%	0.365	0.728	-
Previous infection^a^	15.4%	26.0%	0.196	0.203	-	16.7%	24.7%	0.335	0.836	-
Previous vaccination^a^	59.0%	49.4%	0.327	0.078	-	55.6%	50.6%	0.622	0.631	-
Previous corticoid therapy^a^	15.4%	6.5%	0.123	0.199	-	11.1%	8.6%	0.673	0.801	-
Helminth infection	42.9%	25.0%	0.091	0.252	-	21.4%	36.1%	0.167	0.324	-
Z scores for weight-for-age and gender	−0.1	−0.3	0.800	0.990	-	−0.3	−0.1	0.317	0.858	-
	(−1.0, 0.3)	(−0.9, 0.5)				(−1.0, 0.3)	(−0.9, 0.5)			
Ferritin level (ng/mL)	35.2%	30.6	0.243	0.767	-	29.9	35.4	0.323	0.705	-
	(26.4, 48.4)	(20.8, 44.7)				(22.3, 40.8)	(21.6, 49.9)			
Vitamin D level (ng/mL)	40	35.5	0.097	0.963	-	37.7	35.7	0.736	0.227	-
	(26.8, 59.8)	(23.8, 46.3)				(26.0, 49.1)	(24.4, 48.9)			

TST: Tuberculin Skin Test, QFT: QuantiFERON-TB Gold In-Tube, MTC-score: *Mycobacterium tuberculosis* contact score, BCG: Bacillus Calmette-Guérin.

# Categorical variables expressed as percentage (%) and quantitative variables expressed as mean and interquartile range (IQR).

a In the 12 weeks prior to the study.

b Linear odds ratio.

On analysis at a bivariate level, QFT+ respect to QFT− results showed an association with the MTC-score (U = 984.0, *P* = 0.021) and time of exposure (U = 894.5 *P = *0.024). The logistic regression model showed that the QFT results only depended on the MTC-score (Linear OR = 1.161; CI: 1.010–1.333) (Table 3 and Figure 2).

### Discordant Study Results

The discordance between the tests was analyzed based on the TST results to identify the possible factors that may explain the QFT results.

#### TST+ patients

In the TST+ patients the presence of greater exposure showed a greater possibility of QFT+ results (Linear OR = 1.727; CI: 1.091–2.735) while the presence of intestinal helminthiasis determined a greater possibility of QFT− results (OR = 0.064; CI: 0.004–0.983) (Table 4).

**Table 4:** Multivariate logistic regression according to factors for positive QFT results stratified by TST results.

	TST+			TST−		
	*P* value	Regression	Odds Ratio	*P* value	Regression	Odds Ratio
		Coefficient	(95% CI)		Coefficient	(95% CI)
Time of exposure (months)	0.667		-	0.593		-
MTC-score	0.020	0.547^a^	1.727^b^	0.288		-
		(0.234)	(1.091; 2.735)			
Helminth infection	0.049	−2.752	0.064	0.895		-
		(1.395)	(0.004; 0.983)			
Ferritin level	0.143		-	0.036	−0.045^a^	0.956^b^
					(0.021)	(0.916; 0.997)

TST: Tuberculin Skin Test, QFT: QuantiFERON-TB Gold In-Tube, MTC-score: *Mycobacterium tuberculosis* contact score, BCG: Bacillus Calmette-Guérin.

a logistic regression coefficient related to quantitative variable.

b Linear odds ratio; exponential to the regression coefficient.

Among the TST+ individuals with intestinal heminths, 66.7% were QFT− (Table 5). However, among those who were TST+ but without helminths only 25.0% were QFT−. On analyzing the concordances between the tests according to the presence of intestinal helminths we found an absence of concordance with the presence of helminths with a Kappa index of 0.211 (*P* = 0.214), showing moderate concordance with a Kappa index of 0.471 (*P*<0.001) when there were no helminths.

**Table 5:** Comparison of the main variables according to TST and QFT results.

		MTC-score		Time of exposure		Ferritin		Helminths
Test results	N_1_	Median	IQR	Median	IQR	Median	IQR	% (n/N_2_)
TST+&QFT+	21	12.50	10.25–14.75	4.00	1.00–12.00	34.35	24.15–65.60	25.0 (4/16)
TST+&QFT−	18	11.50	8.00–13.75	6.71	1.00–7.50	35.40	27.15–41.50	66.7 (8/12)
TST−&QFT+	15	10.00	7.00–12.00	2.00	1.00–4.00	26.60	17.10–31.90	16.7 (2/12)
TST−&QFT−	62	9.50	0.00–12.00	1.00	0.00–3.00	31.00	21.00–57.70	27.1 (13/48)

IQR: interquartile range,

N_1_: number of subjects, TST: Tuberculin Skin Test, QFT: QuantiFERON-TB Gold In-Tube, MTC-score: *Mycobacterium tuberculosis* contact score, n: number of subjects with intestinal helminths;

N_2_: number of subjects studied for helminths.

#### TST− patients

In the TST− patients the QFT results were related to the blood ferritin levels; specifically, the lower the level of ferritin the greater the possibility of being QFT+ (Linear OR = 0.956; CI: 0.916–0.997) (Table 4).

**Figure 4:**
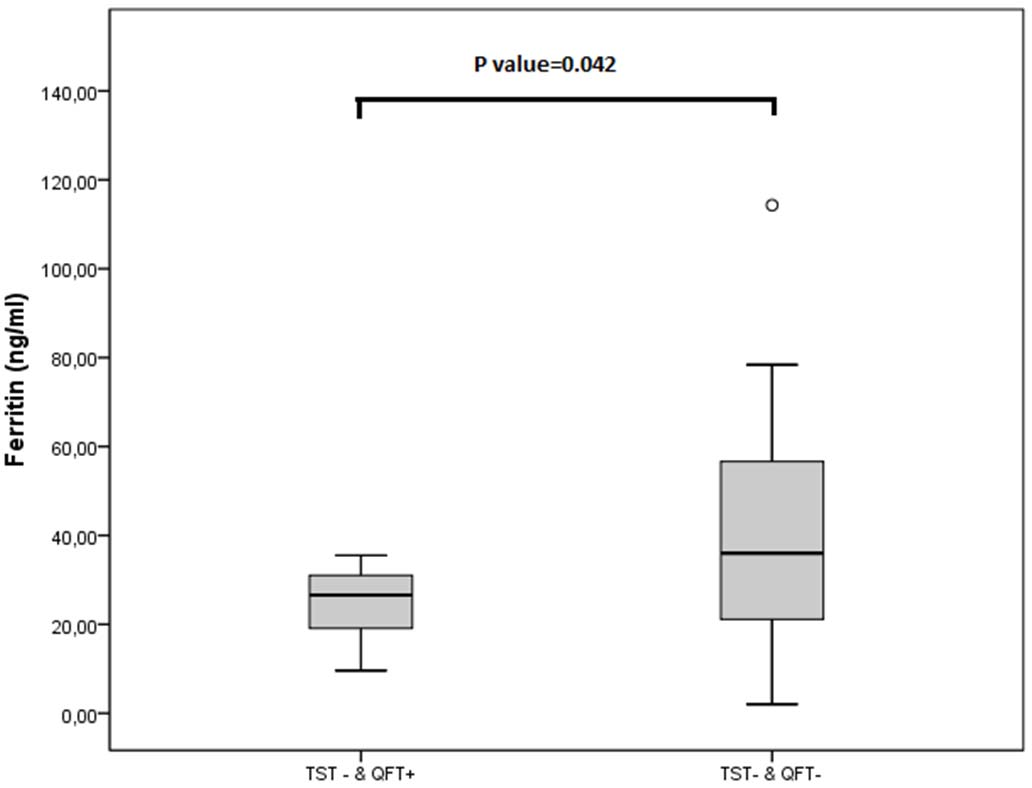
Box-plot of ferritin levels (ng/mL) according to TST−&QFT+ (n = 15) and TST−&QFT− (n = 62) groups. Tuberculin Skin Test (TST) and QuantiFERON-TB Gold in-Tube (QFT).

Of the TST− and QFT+ individuals, 86.7% (13/15) showed ferritin levels lower than 31.9 ng/mL (median of all children studied) (Figure 4). A cut-off point of 37.5 ng/mL was calculated for ferritin with a ROC curve which allowed differentiating the QFT+ from the QFT− individuals in this group, showing a sensitivity of 100% and a specificity of 51%.

## Discussion

The diagnosis of infection by *Mtb* in children is of fundamental importance, especially in regions with a high incidence where BCG vaccination is common. In our study in small children the QFT and TST results were mainly related to the level of exposure to an index case of TB. The discordant results, and thus, the performance of the TST and QFT tests were associated with the presence of intestinal helminths, the length of exposure to an index case and blood ferritin levels.

Although several studies have compared these two diagnostic techniques, the concordances vary widely especially based on the prevalence of TB in the region [14]. Few studies in young children have evaluated the discordant results between the tests [9] and the interpretation of the two tests according to the factors associated with the characteristics of contagion and those which may alter immune response.

### General Concordance and Analysis of the Test Responses

In this study the percentage of positives for TST was 34.8%, being 26.7% for QFT, with fair concordance between the two tests (Kappa = 0.35). We observed that the results of both tests were related to the intensity of exposure, although, as previously reported, the TST was more strongly influenced by exposure than QFT [10], [15]. Another factor we observed was that TST+ results were related to a greater time of exposure while the same was not observed for QFT. This finding is fundamental since young children have probably not been previously exposed to *Mtb* [6] thereby suggesting that in our study two different situations may be found, one with short exposure in which we find an early phase of primary infection by *Mtb* and another with a longer time of exposure showing latent tuberculosis infection (LTBI) secondary to primary infection. This may explain the behavior of the different tests according to the time of exposure [16] due to different immune response in relation to the time of infection observed.

Likewise, we did not observe any association between the TST results and age or the presence of a BCG scar. Thus, previous BCG vaccination or environmental exposure to NTM (in which the probability of weak or strong TST reactions should increase with age due to the greater probability of exposure to NTM) does not, *a priori,* seem to play an important role in TST behavior, similar to what other authors have reported [17], [18], especially in tropical regions [19]. In addition, on analyzing our data the higher rate of TST+ results could be explained; at least in part, by a greater sensitivity or stability over time [20] versus the QFT which may be affected by reversion phenomenon (negativization of the test after positivity) during latent infection [21].

### Discordant Test Results

On analyzing the discordant test results in our study the TST+ group was found to have a greater probability of presenting QFT− results in association with the presence of intestinal helminths. Intestinal helminths may explain up to two thirds of the discordant test results in this group, probably due to an ineffective Th1 immune response leading to a lower secretion of IFN-γ [22] and, thus, a false negativization of the QFT. On quantitative analysis of the values of IFN-γ in the individuals of this group we observed all presented values lower than 0.18 IU/mL. Thus, a lower threshold of positivity for QFT in this population, as suggested by some authors [23], [24], would not explain the discordant results in this group. To our knowledge the association of intestinal helminths and QFT− results has not been previously described. The involvement of the presence of helminths on QFT performance has been previously reported, being associated with a greater percentage of indeterminate results secondary to no reactivity of the mitogen control [25], [26]. This phenomenon may be explained by the immunomodulating capacity of infection by intestinal helminths, producing a strong Th2 immune response and thus, significant suppression of Th1 response [22]. Human and animal studies have shown that individuals with TB and helminthiasis have a reduction in IFN-γ secretion and the number of T CD4 lymphocytes compared to subjects with TB alone [22]. Effective immune response to *Mtb* requires complete Th1 response [27]. Some studies have reported that helminthic infections may facilitate the progression of *Mtb* [28], [29].

This special immunological situation with the presence of helminths may also explain, in part, the QFT reversion phenomena. These reversion phenomena have, to date, been basically related to the clearance of *Mtb* [30], the intermittent expression of the antigens of the RD1 region of *Mtb* during the latent phase [31], variability in laboratory procedures [32] and non-specific variations in IFN-γ levels when the test is repeated in the same individual [21].

On analyzing the discordant test results in the TST− group we observed that low ferritin levels were associated with QFT+ results. Likewise, we found that the TST− group presented lower exposure times (mean = 2.11, median = 1) than the TST+ individuals (mean = 7.32, median = 4). These two facts together, in addition to the fact that small children had probably not been previously exposed to *Mtb,* suggest that these individuals may be in an early phase of primary infection by *Mtb*. It has previously been reported that the antigens used in the Interferon-γ Release Assays ESAT-6 and CPF-10 are secreted mainly in early infection [16], [33], [34] and some studies in animals have related the initial response of primary infection to *Mtb* to a rapid rise in IFN-γ and a fall in ferritin values [35] suggesting a true positivization of the QFT.

Iron (Fe) regulation requires a delicate balance between preventing an excess of Fe favoring survival, multiplication and virulence of *Mtb* [36] and providing sufficient Fe to ensure effective immune response [37]. Mycobacteria competes for the sources of Fe with the host, thus, one strategy of immune response would be to decrease the availability of Fe to the mycobacteria [37], [38]. Ferritin is a fundamental protein for the regulation of Fe, acting as a storage protein and managing the intracellular distribution of this element [39]. The clinical evolution of TB is reportedly worse in humans with both high and low ferritin levels [40].

The results of animal studies have indicated that a decrease in ferritin levels in primary infection is mainly mediated by IFN-γ [35]. This phenomenon is not exclusive of subjects vaccinated with BCG but is more prolonged and more intense over time. Some authors have reported that strong regulation of Fe metabolism could explain, in part, the protector effect of the BCG vaccine against TB [35], [37].

Analysis of our data supports the contention that QFT probably undergoes more rapid conversion (step from negative to positive) after primary infection than the TST and would explain most of the discordant test results in this group as well as reinforce the importance of iron metabolism in the immune response against *Mtb* [41]. This is, to our knowledge, one of the first studies to explain this discordance related to time of exposure, ferritin levels and relating the metabolism of Fe with the initial immune response to primary infection in humans [26], [42].

### Indeterminate QFT Results

The 14% of indeterminate results for QFT in this study suggests a limitation of the usefulness of IGRA in the pediatric TB setting. The proportion of indeterminate results varies greatly in studies in children [43]–[45] and may be associated with technical problems and probably also factors which alter Th1 immune response [46] such as age, the presence of helminths, and immunosuppression [25]. We found no association with any of the variables analyzed in this study or with the results of the TST, probably due to a lack of statistical power.

## Conclusions

The results of the present study strongly suggest the utility of the systematic study of household pediatric contacts for early detection of infection by *Mtb*.

Despite observing a lower concordance than expected, we observed that the main variable which may explain the results of the TST and QFT tests was the level of exposure to an index case. Likewise, the time of exposure was critical in the evaluation and reading of the test results.

On studying the discordant results between the tests we found that the presence of intestinal helminths and a short exposure time explained a large part of these results. For the interpretation of the discordant test results we recommend the study of intestinal helminthiasis (especially in settings of high prevalence) and estimation of the time of exposure and measurement of ferritin levels.

In the case of TST+&QFT− results, the presence of intestinal helminths may produce false negative QFT results due to the negative impact of the helminths on Th1 immune response. In the case of TST−&QFT+ results, low ferritin levels (<37.5 ng/mL) and short time of exposure (<2 months) could indicate initial stages of infection by *Mtb* with possible false negative TST results.

This study suggests the important role of iron metabolism in human immune response in primary infection by *Mtb* and the possibility that ferritin may be a biomarker in this phase [42], [47].
